# Root Canal Treatment After Fixed Prosthodontic Restorations: A Retrospective Observational Study

**DOI:** 10.3390/jcm15135281

**Published:** 2026-07-06

**Authors:** Ebru Arslan, Ceren Gedikli Cengiz, Ibrahim Atakan Cengiz, Selim Erkut, Kamran Gulsahi

**Affiliations:** 1 Department of Prosthodontics, Faculty of Dentistry, Başkent University, Yukarıbahcelievler Street No:26, Çankaya, Ankara 06000, Turkey; atakancengiz23@hotmail.com (I.A.C.); selimerkut@gmail.com (S.E.); 2 Department of Endodontics, Faculty of Dentistry, Başkent University, Ankara 06490, Turkey; cerengediklii8@gmail.com (C.G.C.); kgulsahi@baskent.edu.tr (K.G.)

**Keywords:** root canal therapy, dental porcelain, prosthodontics, retrospective studies

## Abstract

**Background/Objectives:** Root canal treatment (RCT) may be required after full-coverage crown placement in initially vital teeth due to biological and restorative factors affecting pulp vitality. However, the timing and distribution of clinical variables associated with teeth requiring RCT after crown placement remain insufficiently described. The aim of this study was to evaluate this timing and distribution using retrospective data collected between 2011 and 2024. **Methods**: This study included 588 vital teeth treated with metal–ceramic or zirconia crowns between 2011 and 2024. Recorded variables were age, gender, tooth type and location, crown material, bruxism, number of abutment teeth, and time to RCT. Statistical analyses were performed using SPSS v25.0, and as data were not normally distributed (Shapiro–Wilk test), Mann–Whitney U, Kruskal–Wallis, and chi-square or Fisher’s exact tests were used (*p* < 0.05). **Results:** Of the 588 teeth, 36.1% were from male and 63.9% from female patients, and most crowns were metal–ceramic (80.8%), followed by zirconia (19.2%). Significant associations were observed between tooth group and jaw location (*p* < 0.001), number of abutment teeth (*p* < 0.001), and crown material type (*p* < 0.001). RCT was most frequently recorded during the fifth year after crown placement (52.6% of cases), with a mean time of 4.13 ± 1.79 years. Tooth extraction was observed in 5.3% of teeth. **Conclusions**: This study provides descriptive information regarding the distribution and timing of RCT in initially vital teeth following fixed prosthodontic restorations. The findings should be interpreted as a characterization of affected cases rather than as evidence of risk factors or predictors of RCT occurrence.

## 1. Introduction

Full-coverage crowns are a common prosthetic treatment used to restore, protect, and improve the appearance of severely damaged teeth, as well as to provide functional rehabilitation. They are commonly used for large carious lesions, tooth fractures, esthetic issues, and prosthetic needs, such as acting as abutments for fixed dental prostheses [1]. Although full crowns help ensure long-term structural integrity, they require significant tooth preparation, which can expose the pulp to mechanical, thermal, and chemical damage. High-speed instruments, dehydration, marginal leakage, and bacteria from previous restorations can compromise pulp health, particularly when protective dentine barriers are thinned or removed [2,3,4].

Teeth with deep restorations or a history of recurrent caries may already exhibit subclinical inflammation. When preparing such teeth for crowns, the accumulated stress can accelerate pulp degeneration, progressing from reversible inflammation to irreversible pulpitis or necrosis [5,6]. Furthermore, the use of eugenol-free temporary cements, an improper temporary fit, and delayed definitive cementation can exacerbate pulp irritation. Studies have shown that these effects may manifest clinically as pain and sensitivity, or sometimes require root canal treatment (RCT) years after crown placement [7].

Recent prosthodontic literature indicates that complications associated with tooth-supported fixed restorations tend to accumulate over time and may involve biological, technical, and esthetic domains simultaneously. Long-term clinical studies have demonstrated that restoration age, material characteristics, oral hygiene, parafunctional habits, and patient-related factors may influence the occurrence of prosthodontic complications. However, most available studies have focused on restoration survival and overall complication rates rather than specifically evaluating the timing of RCT in initially vital teeth following crown placement [8,9,10]. Won and Berlin-Broner [11] demonstrated that the presence of multiple prior restorations increases the likelihood of pulp failure in patients with vital teeth. These findings suggest that pulpal complications may occur following crown placement in initially vital teeth.

Given these concerns, preservation of pulp vitality remains a central objective of contemporary restorative and endodontic treatment [12]. The long-term prognosis of a restored tooth depends not only on the integrity of the prosthetic restoration but also on the maintenance of pulpal health [13]. Even when teeth remain asymptomatic at the time of crown placement, cumulative biological insults associated with previous restorations, caries, tooth preparation, and restorative procedures may compromise pulpal blood supply and reparative capacity over time. As a result, pulpal degeneration may progress gradually and remain clinically undetectable until irreversible pulpitis or pulp necrosis develops [14].

Endodontic diagnosis is based on the combined interpretation of patient history, clinical examination, pulp sensibility testing, and radiographic findings. Recent recommendations from the European Society of Endodontology (ESE) and the American Association of Endodontists (AAE) have emphasized the importance of standardized diagnostic terminology for conditions such as irreversible pulpitis and pulp necrosis, thereby improving consistency in clinical decision making and research reporting [15,16].

The biological response of the pulp following crown placement is complex and may be influenced by both treatment- and patient-related factors. Thermal trauma during preparation, dentin dehydration, microleakage, bacterial penetration, and occlusal stress may contribute to progressive pulpal injury. Importantly, these processes do not always result in immediate symptoms, and endodontic complications may emerge several years after restorative treatment [5,7,17]. Consequently, evaluating the temporal pattern of RCT occurrence following crown placement may provide valuable information regarding the long-term biological consequences of fixed prosthodontic therapy.

Several clinical and restorative variables, including tooth type, crown material, adhesive cement, patient age, and parafunctional habits such as bruxism, have been inconsistently associated with endodontic outcomes in the literature. Among these variables, crown material has received particular attention because of its potential influence on the biological performance and long-term prognosis of fixed prosthodontic restorations. Although recent studies have reported favorable biological outcomes and acceptable medium-term survival rates for both zirconia and metal–ceramic restorations, limited information is available regarding the time interval between crown placement and the subsequent need for RCT [10,18]. Most investigations have focused on restoration survival, technical complications, or general biological outcomes, whereas the timing of endodontic intervention has received considerably less attention. Consequently, the temporal pattern of RCT occurrence following crown placement remains insufficiently characterized in the current literature.

This retrospective study aimed to evaluate the time interval between crown placement and RCT and to describe the distribution of clinical and restorative variables among initially vital teeth that subsequently required RCT.

## 2. Materials and Methods

### 2.1. Study Design

This study includes the clinical data of adult patients who underwent crown treatment at the Department of Prosthodontics at Baskent University’s Faculty of Dentistry between 2011 and 2024, followed by RCT at the institution’s Department of Endodontics. All patients provided routine institutional informed consent upon admission for dental treatment at Baskent University Faculty of Dentistry. As this study was conducted retrospectively through review of existing clinical records, no additional written informed consent was obtained, and data collection was completed in January 2025. The Institutional Review Board and Ethical Committee of Baskent University approved this retrospective study (Approval No: D-KA25/02, date: 20 January 2025), which was conducted in accordance with the principles of the Declaration of Helsinki, and it was also conducted and reported in accordance with the Strengthening the Reporting of Observational Studies in Epidemiology (STROBE) Statement (Supplementary Material S1) [19].

Patients who had undergone crown placement at the Department of Prosthodontics and subsequently required RCT were identified through retrospective review of institutional electronic patient records, including clinical notes and anamnesis data from the Department of Endodontics. Treatment records from the Departments of Prosthodontics and Endodontics were matched using the institution’s electronic health record system. This initial search yielded 1910 patients who had received treatment in both departments, regardless of whether prosthodontic or endodontic treatment had been received first. The clinical records and anamnesis data of these patients were then reviewed individually to identify cases in which root canal treatment had been performed following crown placement. After applying the predefined inclusion and exclusion criteria, the final study sample comprised 478 patients and 588 teeth.

### 2.2. Patient Selection

The inclusion criteria comprised adult patients who underwent fixed prosthetic treatment involving full crowns or bridges and who subsequently required RCT. Only teeth that were vital at the time of crown placement and had no documented history of spontaneous pain, prolonged thermal sensitivity, previous RCT, periapical pathology, radiographic signs of pulpal or periapical disease, or a diagnosis of irreversible pulpitis before restoration were included. Additionally, cases involving metal–ceramic or zirconia crown restorations were considered eligible for analysis.

Endodontic risk before restoration was defined as the presence of spontaneous pain, prolonged sensitivity to thermal stimuli, previous endodontic treatment, periapical pathology, radiographic signs of pulpal or periapical disease, or a diagnosis of irreversible pulpitis before crown placement [15,16].

**Figure 1:**
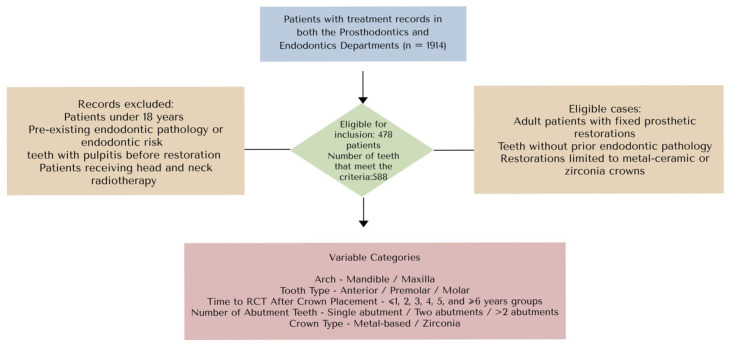
Flowchart of Patient Selection, Eligibility Criteria, and Variable Classification.

Patients under 18 years of age, presenting with any of the above-mentioned endodontic risk factors before restoration, and undergoing radiotherapy for head and neck cancer were excluded (Figure 1).

### 2.3. Clinical and Radiographic Evaluation

The indication for RCT was determined by the attending endodontist based on clinical symptoms, pulp sensibility testing, and radiographic findings consistent with irreversible pulpitis or pulp necrosis, in accordance with established diagnostic criteria [15,16].

No standardized questionnaires or validated assessment forms were used in this study. All variables were obtained retrospectively from institutional electronic patient records, clinical documentation, radiographic examinations, and prosthetic treatment records.

The collected data included patient demographic characteristics (age and gender), the type and location of the treated tooth (anterior, premolar, or molar), crown material (metal–ceramic or zirconia), presence of bruxism, number of abutment teeth, the interval between crown placement and RCT, and the final clinical outcome of the tooth following RCT, including whether extraction was required during follow-up. Bruxism was recorded based on patient self-report and clinical findings documented in the patient records.

For descriptive purposes, the elapsed time between crown placement and RCT was categorized according to the year in which RCT was performed after crown placement. Teeth requiring RCT within the first year after crown placement were assigned to the ≤1-year category. Cases in which RCT was performed between 1 and 2 years, 2 and 3 years, 3 and 4 years, and 4 and 5 years after crown placement were assigned to the 2-year, 3-year, 4-year, and 5-year categories, respectively. Cases occurring more than 5 years after crown placement were grouped into the ≥6-year category. Teeth that required RCT immediately after tooth preparation or shortly after crown placement were also included in the ≤1-year category.

### 2.4. Statistical Analysis

A total of 588 teeth were included in the study. As this was a retrospective observational study, all eligible cases meeting the inclusion criteria during the study period were included in the analysis. No missing data were identified, and only cases with complete records for all study variables were analyzed.

Data analysis was performed using the SPSS v25.0 (SPSS; IBM Corp., Armonk, NY, USA) statistical program. The mean ± standard deviation and the median (minimum–maximum) were calculated for numerical variables, and the frequency and percentage values were calculated for categorical variables. The normality of the distribution of numerical variables was assessed using the Shapiro–Wilk test, but as the normal distribution assumption was not met for all numerical variables, non-parametric methods were used for comparisons. The Mann–Whitney U test was used for comparisons between two independent groups and the Kruskal–Wallis test for those between three or more groups. Relationships between categorical variables were analyzed using the Pearson chi-squared test. Where necessary, Fisher’s exact test and continuity correction were applied. The significance level was set at *p* < 0.05 for all analyses.

## 3. Results

### 3.1. Characteristics of the Study Sample

A total of 588 teeth restored with fixed prosthetic restorations and subsequently requiring RCT during follow-up were included in the study, among which 36.1% belonged to male patients and 63.9% to female patients. The median patient age was 59 years. Regarding jaw location, 42.5% of the teeth were in the mandible and 57.5% in the maxilla. Anterior teeth represented the largest tooth group (35.2%), followed by molars (33.0%) and premolars (31.8%). Metal–ceramic crowns constituted 80.8% of restorations, whereas zirconia crowns accounted for 19.2%, and most restorations were supported by more than two abutments (72.4%). Tooth extraction following RCT occurred in 31 teeth (5.3%), and the mean time between crown placement and extraction was 4.03 ± 3.21 years. Bruxism was identified in 10 cases (1.7%) (Table 1).

### 3.2. Distribution of Tooth Groups According to Clinical Variables

Anterior teeth were predominantly located in the maxilla (72.9%), whereas molars were more frequently found in the mandible (54.1%), and premolars showed a relatively balanced distribution between the two jaws (52.4% maxilla vs. 47.6% mandible). A significant association was observed between tooth group and jaw location (*p* < 0.001). Restorations supported by more than two abutments represented the most common configuration in anterior (76.8%) and premolar teeth (80.2%), whereas single-abutment restorations were proportionally more frequent in molars (30.4%). The distribution of abutment configurations differed significantly among the tooth groups (*p* < 0.001) (Table 2).

A significant association was also found between tooth group and crown material type (*p* < 0.001). Zirconia crowns were proportionally more common in anterior teeth (27.5%), whereas metal–ceramic crowns predominated in molars (88.1%). When crown materials were evaluated according to gender, zirconia restorations were proportionally more frequent among female than male patients (23.7% vs. 11.3%; Pearson chi-square test, χ^2^ = 13.318, df = 1, *p* < 0.001) (Table 3).

### 3.3. Time Interval Between Crown Placement and Root Canal Treatment

The mean time elapsed until RCT was 4.13 ± 1.79 years. No statistically significant difference was found in time to RCT among tooth groups (Kruskal–Wallis test, *p* = 0.270), crown materials (Mann–Whitney U test, *p* = 0.648), or jaw locations (Mann–Whitney U test, *p* = 0.065) (Table 4).

RCT was most frequently observed during the fifth year after crown placement, accounting for 52.6% of all cases. Early RCT within the first year after crown placement was observed in 17.3% of teeth (Table 5).

Although maxillary teeth demonstrated a slightly longer mean time to RCT than mandibular teeth, the difference did not reach statistical significance. Similarly, comparable time intervals were observed between metal–ceramic and zirconia restorations.

## 4. Discussion

The present study provides descriptive information regarding the timing and distribution of RCT following fixed prosthodontic treatment in initially vital teeth. Although causal inferences cannot be drawn, the findings suggest that pulpal complications may occur several years after crown placement, emphasizing the importance of long-term follow-up and careful patient monitoring after definitive prosthetic treatment.

Due to the very low prevalence of bruxism observed in this study, at only 1.7% of cases, no meaningful interpretation could be made regarding its potential role in RCT occurrence. Previous studies evaluating fixed prosthodontic complications have reported substantially higher frequencies of bruxism when self-reported questionnaires and clinical examinations were used [20]. This suggests that parafunctional habits may be underreported in retrospective studies based primarily on clinical records. Traumatic occlusion has been noted to negatively affect pulp tissue by causing microleakage at the dentin–restoration interface, which may lead to apical inflammation in the long term [21,22,23].

Previous prospective evidence has shown that pulp necrosis developed in 9% of teeth following crown preparation and electrical pulp testing and occurred significantly more frequently in previously restored teeth [17]. It was reported that necrosis occurred only in mandibular anterior teeth. In our study, previous restorations were not evaluated, and maxillary anterior teeth represented the largest subgroup among teeth requiring RCT. By contrast, Won et al. [11] reported that molars represented the largest proportion of teeth requiring RCT (55%). When evaluating the distribution of tooth groups according to abutment number, single-abutment restorations were proportionally more frequent in molars (30.4%).

The literature indicates that single-tooth abutment restorations, especially in the posterior region, may damage the dentin–pulp complex due to increased occlusal stress and may contribute to microleakage [24]. When examining the relationship between the tooth group and the jaw location, it was observed that the vast majority (72.9%) of anterior teeth undergoing RCT were in the maxilla. This situation may be related to the more frequent application of crowns in this region due to esthetic expectations [10]. Conversely, the high proportion of mandibular molars undergoing RCT (54.1%) may be due to these teeth being subject to high chewing forces and having a more complex anatomy. They also require restorative procedures more frequently due to greater loss of material in the posterior region [25].

In our study, RCT was most frequently observed during the fifth year after crown placement. Previous long-term follow-up studies have reported that RCT is most frequently observed during the mid-term follow-up period after fixed prosthodontic treatment, particularly around the fifth year [26]. Furthermore, in our study, it is noteworthy that 17.3% of teeth required RCT within the first year after crown placement in all tooth groups (anterior/premolar/molar). These findings suggest that pulpal complications may occur both in the early and late periods following restorative procedures. RCT performed within the first year may be related to the presence of subclinical pulp damage prior to restoration, aggressive preparation techniques, inadequate temporary restorations, or irritant factors during the cementation process [6]. Factors such as thermal trauma, excessive drying, or violation of biological width during preparation can lead to pulpitis or necrosis in the early period. By contrast, the higher frequency of RCT observed during the fifth year may be associated with microleakage developing over time, deterioration of marginal adaptation, secondary caries, or the long-term negative impact of occlusal stress accumulation on pulp vitality [27].

Previous findings indicate the presence of endodontic complications in teeth with fixed prostheses over the long term. For example, in a 10-year study, the incidence of periapical lesions in 222 vital teeth supported by fixed prostheses was reported to be 17% [28]. Goodacre et al. [25] found that RCT was required in 3% of 823 teeth with single crowns and 11% of 2514 teeth with bridge abutments; these results indicate that the need for RCT is higher in bridge abutments than in single crowns. Similarly, our findings showed that bridge abutments were more commonly represented among teeth requiring RCT than single crowns, which is consistent with previous research. Another study reported that 9.6% of the 520 teeth used as abutments for removable partial dentures required RCT, 0.6% required retreatment, and the average treatment duration was 3.7 years [29]. In our study, the average duration was found to be approximately 4 years. Additionally, Valderhaug et al. [27] evaluated 291 vital crowned teeth in a 25-year follow-up; pulp vitality loss was observed in only 10% of them, and fewer pulp complications were reported in short bridges. In long bridges, factors such as increased preparation depth, seating problems, and hygiene difficulties were suggested to increase pulp complications.

Clinical studies have reported favorable biological outcomes and relatively low frequencies of endodontic complications among teeth restored with zirconia crowns during medium-term follow-up periods [10,18]. According to the literature, only 4% of posterior teeth required RCT during a five-year follow-up period, in both the anterior and posterior groups [18]. In our study, zirconia crowns were present in 19.2% of teeth that underwent RCT, and they were proportionally more common among anterior teeth requiring RCT, while they were more common in molars with metal-supported crowns. This finding may be explained by the preference for zirconia restorations in the anterior region because of esthetic demands and reduced dentin thickness. By contrast, the predominance of metal-supported crowns in molars may be related to higher occlusal loads and increased susceptibility to microleakage and pulpal complications [24]. Furthermore, in our study, the number of female patients who underwent RCT on zirconia crowns was higher. This situation may be related to the greater esthetic needs in the anterior region and the higher frequency of visits to the dentist among women. However, a 10-year study examining over 88,000 crowns found that all-metal crowns had the lowest rate of biological complications, followed by metal-supported porcelain crowns and then all-ceramic crowns in terms of the need for RCT [30]. Nevertheless, it should be noted that various ceramic systems, such as older-generation weak porcelains, may be included in the ‘all-ceramic’ category of crowns, which could affect the results. In the current follow-up study, monolithic zirconia and metal–ceramic single crowns exhibited comparable success rates. In the 3–6 year follow-up, the biological complication rate was reported as 4.4% in the zirconia group and 8.5% in the metal–ceramic group. Although this difference was not statistically significant, zirconia crowns showed a lower tendency [10].

Zirconia crowns may provide favorable biological properties for preserving pulp health due to their high biocompatibility and low thermal conductivity [31]. Low thermal conductivity reduces thermal trauma; the precise marginal fit achieved with digital impressions minimizes microleakage. Furthermore, since they can be applied with less tooth structure removal, they contribute to maintaining the distance to the pulp chamber [32,33].

This study has several limitations. First, the presence of previous restorations was not evaluated, and the absence of a pre-crown vitality test makes it difficult to definitively determine the causes of pulpal complications that developed after crown placement. In addition, the restorations were prepared and delivered by different clinicians, and several potential confounding factors—including systemic conditions, oral hygiene status, regular dental attendance, operator-related variables, preparation depth, temporary restoration quality, and cementation protocols—could not be standardized or controlled, because of the retrospective study design. Another important limitation is that the study dataset was generated from patients with records of both crown placement and subsequent RCT. Consequently, the total number of crowned teeth treated during the study period could not be reliably determined, and incidence rates or risk estimates could not be calculated. Furthermore, only teeth that subsequently required RCT were included, without a comparison group of crowned teeth that maintained pulp vitality. Therefore, the findings should be interpreted as a descriptive characterization of affected cases rather than evidence of causal relationships, predictors, or risk factors associated with RCT occurrence. As data were obtained from institutional records, patients who underwent RCT at external centers following crown placement at our institution may not have been captured, potentially leading to an underestimation of the true frequency of post-restorative endodontic complications. Finally, as this was a single-center retrospective study conducted at a university dental clinic, the generalizability of the findings to other clinical settings and patient populations may be limited. Future prospective controlled studies are needed to better clarify the factors associated with long-term pulp vitality following fixed prosthodontic treatment.

## 5. Conclusions

This retrospective observational study provides descriptive information regarding the timing and distribution of RCT in initially vital teeth following fixed prosthodontic restorations. RCT was most frequently observed during the fifth year after crown placement, although a considerable proportion of cases occurred within the first year, suggesting that pulpal complications may develop during both the early and long-term phases after restorative treatment.

Among the teeth that underwent RCT, anterior, premolar, and molar teeth were represented in broadly similar proportions, whereas restorations supported by more than two abutments and metal–ceramic crowns predominated. However, because the study included only affected teeth and lacked a comparison group of crowned teeth that maintained pulp vitality, the findings should be interpreted as a characterization of affected cases rather than as evidence of causal relationships, predictors, or risk factors associated with RCT occurrence.

Within the limitations of this retrospective design, the present findings contribute to the understanding of the temporal distribution of endodontic complications following fixed prosthodontic treatment. Future prospective controlled studies are needed to further clarify the factors associated with long-term pulp vitality and endodontic outcomes in restored teeth.

## Figures and Tables

**Table 1:** Demographic and clinical characteristics of the included teeth (n = 588).

Variable	Category	*n*	%	Median Age
**Gender**	Male	212	36.1	61.5
	Female	376	63.9	58.0
**Age**	Total	588	-	59.0
**Jaw Location**	Mandible	250	42.5	–
	Maxilla	338	57.5	–
**Tooth Type**	Anterior	207	35.2	–
	Premolar	187	31.8	–
	Molar	194	33.0	–
**Number of Abutment Teeth**	Single abutment	112	19.0	–
	Two abutments	50	8.5	–
	>2 abutments	426	72.4	–
**Crown Type**	Metal-ceramic	475	80.8	
	Zirconia	113	19.2	
**Bruxism**	No	578	98.3	–
	Yes	10	1.7	–
**Tooth Extraction**	No	557	94.7	–
	Yes	31	5.3	–

Percentages were calculated from the total sample (*n* = 588). Continuous variables are presented as mean ± standard deviation (SD).

**Table 2:** Distribution of jaw location and abutment configuration according to tooth group (*n* = 588).

Tooth Group	Maxilla *n* (%)	Mandible *n* (%)	Single Abutment *n* (%)	Two Abutments *n* (%)	>2 Abutments *n* (%)	Total
**Anterior**	151 (72.9)	56 (27.1)	31 (15.0)	17 (8.2)	159 (76.8)	207
**Premolar**	98 (52.4)	89 (47.6)	22 (11.8)	15 (8.0)	150 (80.2)	187
**Molar**	89 (45.9)	105 (54.1)	59 (30.4)	18 (9.3)	117 (60.3)	194
**Total**	338 (57.5)	250 (42.5)	112 (19.0)	50 (8.5)	426 (72.4)	588

Jaw location vs. tooth group: χ^2^ = 32.919, df = 2, *p* < 0.001 (Pearson chi-square test). Abutment number vs. tooth group: χ^2^ = 26.419, df = 4, *p* < 0.001 (Pearson chi-square test).

**Table 3:** Distribution of crown type according to gender and tooth group.

	Metal-Ceramic *n* (%)	Zirconia *n* (%)	Total (*n*)	*p* Value
**Gender**				
Male	188 (88.7%)	24 (11.3%)	212	<0.001
Female	287 (76.3%)	89 (23.7%)	376	
**Tooth Group**				
Anterior	150 (72.5%)	57 (27.5%)	207	<0.001
Premolar	154 (82.4%)	33 (17.6%)	187	
Molar	171 (88.1%)	23 (11.9%)	194	
Total	475 (80.8%)	113 (19.2%)	588	

*p* < 0.001 (Pearson chi-square test).

**Table 4:** Time elapsed until root canal treatment according to clinical variables.

Variable	Category	*n*	Mean ± SD (Years)	Median	Min–Max	*p*-Value
Tooth group	Anterior	207	4.28 ± 1.83	5	1–11	0.270 ^a^
	Premolar	187	4.04 ± 1.79	5	1–8	
	Molar	194	4.05 ± 1.77	5	1–7	
Crown material	Metal	475	4.15 ± 1.76	5	1–11	0.648 ^b^
	Zirconia	113	4.03 ± 1.95	5	1–6	
Jaw location	Mandible	250	3.97 ± 1.83	5	1–9	0.065 ^b^
	Maxilla	338	4.25 ± 1.76	5	1–11	

Continuous variables are presented as mean ± standard deviation (SD), median, and minimum–maximum values. ^a^ Kruskal–Wallis test. ^b^ Mann–Whitney U test.

**Table 5:** Distribution of time to RCT according to tooth group (n = 588).

Tooth Group	≤1 Year *n* (%)	2 Years *n* (%)	3 Years *n* (%)	4 Years *n* (%)	5 Years *n* (%)	≥6 Years *n* (%)	Total
**Anterior**	31 (15.0)	16 (7.7)	12 (5.8)	2 (1.0)	108 (52.2)	38 (18.4)	207
**Premolar**	35 (18.7)	15 (8.0)	7 (3.7)	8 (4.3)	96 (51.3)	26 (13.9)	187
**Molar**	36 (18.6)	15 (7.7)	8 (4.1)	5 (2.6)	105 (54.1)	25 (12.9)	194
**Total**	102 (17.3)	46 (7.8)	27 (4.6)	15 (2.6)	309 (52.6)	89 (15.1)	588

Note: Time categories represent the follow-up year in which RCT was performed after crown placement. The 2-, 3-, 4-, and 5-year categories correspond to cases occurring between 1–2, 2–3, 3–4, and 4–5 years; cases occurring after 5 years were grouped into the ≥6-year category.

## Data Availability

The data presented in this study are available from the corresponding author upon reasonable request. The data are not publicly available due to institutional and privacy restrictions.

## Supplementary Materials

The following supporting information can be downloaded at: https://www.mdpi.com/article/10.3390/jcm15135281/s1, STROBE Statement—checklist of items that should be included in reports of observational studies.
