# Supplementary material for: Root Canal Treatment After Fixed Prosthodontic Restorations: A Retrospective Observational Study
Source: J Clin Med. 2026 Jul 6;15(13):5281. doi: 10.3390/jcm15135281 (PMC13362701; doi:10.3390/jcm15135281)
Supplement: Supplementary file 1 [file jcm-15-05281-s001.zip › jcm-4382964-supplementary.pdf]

STROBE Statement—checklist of items that should be included in reports of observational studies

	Item No.	Recommendation	Page No.	Relevant text from manuscript
**Title and abstract**	1	(*a*) Indicate the study's design with a commonly used term in the title or the abstract	1	"Root Canal Treatment After Fixed Prosthodontic Restorations: A Retrospective Observational Study"
		(*b*) Provide in the abstract an informative and balanced summary of what was done and what was found	1	Structured Abstract (Background/Objectives, Methods, Results, Conclusions)
		**Introduction**		
Background/rationale	2	Explain the scientific background and rationale for the investigation being reported	2–3	Paragraphs describing pulpal complications after crown placement, pulp vitality preservation, endodontic diagnosis, and the rationale for evaluating time to RCT.
Objectives	3	State specific objectives, including any prespecified hypotheses	3	This retrospective study aimed to evaluate the time interval between crown placement and RCT and to describe the distribution of clinical and restorative variables among initially vital teeth that subsequently required RCT.
		**Methods**		
Study design	4	Present key elements of study design early in the paper	4	"This retrospective study..." "This study was conducted and reported in accordance with the STROBE Statement."

Setting	5	Describe the setting, locations, and relevant dates, including periods of recruitment, exposure, follow-up, and data collection	4	Department of Prosthodontics and Department of Endodontics, Başkent University Faculty of Dentistry; study period 2011–2024; data collection completed in January 2025.
Participants	6	(*a*) *Cohort study*—Give the eligibility criteria, and the sources and methods of selection of participants. Describe methods of follow-up<br>*Case-control study*—Give the eligibility criteria, and the sources and methods of case ascertainment and control selection. Give the rationale for the choice of cases and controls<br>*Cross-sectional study*—Give the eligibility criteria, and the sources and methods of selection of participants	4–5	Description of record matching, identification of 1910 patients treated in both departments, eligibility screening, inclusion criteria, exclusion criteria, final sample of 478 patients and 588 teeth.
		(*b*) *Cohort study*—For matched studies, give matching criteria and number of exposed and unexposed<br>*Case-control study*—For matched studies, give matching criteria and the number of controls per case	Not applicable (no matching design).	
Variables	7	Clearly define all outcomes, exposures, predictors, potential confounders, and effect modifiers. Give diagnostic criteria, if applicable	5	Age, gender, tooth type, jaw location, crown material, bruxism, number of abutment teeth, time to RCT, extraction outcome.
Data sources/ measurement	8*	For each variable of interest, give sources of data and details of methods of assessment (measurement). Describe comparability of assessment methods if there is more than one group	5-6	Variables obtained from institutional electronic health records, clinical documentation, radiographic examinations, and prosthetic treatment records.
Bias	9	Describe any efforts to address potential sources of bias	13-14	Limitations section discussing

				retrospective design, absence of comparison group, unavailable denominator population, possible underreporting, and external treatment records.
Study size	10	Explain how the study size was arrived at	6	"As this was a retrospective observational study, all eligible cases meeting the inclusion criteria during the study period were included in the analysis."

Continued on next page

Quantitative variables	11	Explain how quantitative variables were handled in the analyses. If applicable, describe which groupings were chosen and why	5-6	Description of age and time variables and categorization of time to RCT (≤1, 2, 3, 4, 5, and ≥6 years).
Statistical methods	12	(*a*) Describe all statistical methods, including those used to control for confounding	6	Shapiro–Wilk, Mann–Whitney U, Kruskal–Wallis, Pearson chi-square, Fisher's exact test.
		(*b*) Describe any methods used to examine subgroups and interactions	7-9	Analyses according to tooth group, crown material, jaw location, and abutment configuration.
		(*c*) Explain how missing data were addressed	6	"No missing data were identified..."
		(*d*) *Cohort study*—If applicable, explain how loss to follow-up was addressed<br>*Case-control study*—If applicable, explain how matching of cases and controls was addressed<br>*Cross-sectional study*—If applicable, describe analytical methods taking account of sampling strategy	Not applicable.	
		(*e*) Describe any sensitivity analyses	Not applicable.	
**Results**				
Participants	13*	(a) Report numbers of individuals at each stage of study—eg numbers potentially eligible, examined for eligibility, confirmed eligible, included in the study, completing follow-up, and analysed	4–5, Figure 1	1910 patients identified → eligibility assessment → 478 patients and 588 teeth included.
		(b) Give reasons for non-participation at each stage	5, Figure 1	Exclusion criteria described in Figure 1 and Methods section.
		(c) Consider use of a flow diagram	Figure 1	Flowchart of Patient Selection, Eligibility Criteria, and Variable Classification.
Descriptive data	14*	(a) Give characteristics of study participants (eg demographic, clinical, social) and information on exposures and potential confounders	7–8	Table 1 and accompanying Results text.
		(b) Indicate number of participants with missing data for each variable of interest	6	"No missing data were identified..."

		(c) *Cohort study*—Summarise follow-up time (eg, average and total amount)	Not applicable.	
Outcome data	15*	*Cohort study*—Report numbers of outcome events or summary measures over time		
		Case-control study—Report numbers in each exposure category, or summary measures of exposure		
		Cross-sectional study—Report numbers of outcome events or summary measures	*8-10*	*Tables 2–5 and corresponding Results text.*
Main results	16	(*a*) Give unadjusted estimates and, if applicable, confounder-adjusted estimates and their precision (eg, 95% confidence interval). Make clear which confounders were adjusted for and why they were included	8-10	Associations between tooth group, jaw location, crown material, and abutment configuration; time-to-RCT findings.
		(*b*) Report category boundaries when continuous variables were categorized	8-13	
		(*c*) If relevant, consider translating estimates of relative risk into absolute risk for a meaningful time period		Not applicable (no risk estimates).

Continued on next page

Other analyses	17	Report other analyses done—eg analyses of subgroups and interactions, and sensitivity analyses		
**Discussion**				
Key results	18	Summarise key results with reference to study objectives	10–13	Discussion section paragraph 1
Limitations	19	Discuss limitations of the study, taking into account sources of potential bias or imprecision. Discuss both direction and magnitude of any potential bias	13-14	Limitations section.
Interpretation	20	Give a cautious overall interpretation of results considering objectives, limitations, multiplicity of analyses, results from similar studies, and other relevant evidence	10–14	Discussion section and Conclusion.
Generalisability	21	Discuss the generalisability (external validity) of the study results	14	"As this was a single-centre retrospective study conducted at a university dental clinic, the generalisability of the findings..."
**Other information**				
Funding	22	Give the source of funding and the role of the funders for the present study and, if applicable, for the original study on which the present article is based	15	"This research was supported by the Başkent University Research Fund (Project No. D-KA25/02)."

*Give information separately for cases and controls in case-control studies and, if applicable, for exposed and unexposed groups in cohort and cross-sectional studies.

Note: An Explanation and Elaboration article discusses each checklist item and gives methodological background and published examples of transparent reporting. The STROBE checklist is best used in conjunction with this article (freely available on the Web sites of PLoS Medicine at http://www.plosmedicine.org/, Annals of Internal Medicine at http://www.annals.org/, and Epidemiology at http://www.epidem.com/). Information on the STROBE Initiative is available at www.strobe-statement.org.
